# Predicting coronary artery lesions in Kawasaki disease: a nomogram based on LASSO regression feature selection

**DOI:** 10.1186/s12911-026-03637-4

**Published:** 2026-06-16

**Authors:** Jing Su, Ying Bai, Hua Wang, Yingying Ma, Yulei Zhai, Yinghui Guo

**Affiliations:** 1 Department of Laboratory Medicine, Children’s Hospital of Hebei Province, Shijiazhuang, China; 2 Hebei Provincial Clinical Research Center for Child Health and Disease, Shijiazhuang, China; 3 Department of Pediatrics, Hebei Provincial Key Laboratory for Pediatric Cardiovascular Medicine, Key Medical Specialty of Hebei Province, Children’s Hospital of Hebei Province, Shijiazhuang, China; 4 Department of Critical Care Medicine, Children’s Hospital of Hebei Province, Shijiazhuang, China

**Keywords:** Kawasaki disease, Coronary artery lesions, Nomogram, Machine learning

## Abstract

**Background:**

Coronary artery lesions (CALs) represent the most serious complications of Kawasaki disease (KD), and the early identification of children at high risk for CALs is critical for guiding treatment strategies. This study aimed to develop a nomogram-based model to predict the risk of CALs in children with KD.

**Methods:**

This retrospective analysis enrolled 255 children diagnosed with KD between January 2024 and May 2025. Feature selection was performed using the least absolute shrinkage and selection operator regression. Variables with non-zero coefficients were subsequently incorporated into multivariable logistic regression to identify risk factors associated with CALs. A nomogram was constructed based on these predictors, and internal validation was performed using the bootstrap resampling method. The performance of the model was assessed using receiver operating characteristic (ROC) curves, calibration curves, the Hosmer–Lemeshow goodness-of-fit test, and decision curve analysis (DCA).

**Results:**

The incidence of CALs in children with KD was 29.4% (75/255). Our results identified age, clinical type, platelet count (PLT), and thrombin time (TT) as independent risk factors for CALs in children with KD. The area under the ROC curve of the model was 0.807 (95% confidence interval [CI] = 0.750–0.860). The calibration curves and the Hosmer–Lemeshow goodness-of-fit test revealed good consistency between the predicted probabilities and observed outcomes, and DCA confirmed that the model had good clinical utility.

**Conclusions:**

We developed and internally validated a nomogram-based predictive model based on age, clinical type, PLT, and TT that may facilitate the early identification of children with KD at high risk of developing CALs.

**Supplementary Information:**

The online version contains supplementary material available at 10.1186/s12911-026-03637-4.

## Background

Kawasaki disease (KD) is an acute systemic vasculitis that predominantly affects children younger than 5 years [1]. The diagnosis of KD is primarily based on criteria established by the American Heart Association (AHA) [2]. The incidence of KD varies considerably across the globe. It is highest in Japan, reaching 309 per 100,000 children younger than 5 years in 2016 [3], compared with 19.8 per 100,000 children in the United States [4]. KD has been considered the most common cause of acquired heart disease in children in developed countries, and medium-sized blood vessels, especially the coronary arteries, are most commonly affected [5].

Coronary artery lesions (CALs), which include coronary artery dilatation, coronary artery aneurysm, stenosis, and fistula, represent the most severe complications of KD [6]. Although aspirin combined with intravenous immunoglobulin (IVIG) treatment effectively reduces the incidence of CALs in children with KD, 4%–6% of patients still experience CALs, increasing their risk of cardiovascular events [7]. The early identification of children with KD at risk of developing CALs remains crucial to permit timely individualized treatment and reduce the risk of cardiovascular complications.

To date, several clinical prediction models have been developed to predict the risk of CALs in children with KD, incorporating various demographic and laboratory indicators [8–11]. Nevertheless, the variable selection process in these models is primarily based on conventional univariate or multivariate analyses, which have limitations in handling multicollinearity and overfitting among variables [12]. Machine learning (ML) algorithms, such as least absolute shrinkage and selection operator (LASSO) regression and the Boruta algorithm, excel at capturing complex nonlinear relationships and processing high-dimensional data [13]. These algorithms can address the limitations of conventional statistical models, enabling the identification of key predictors and development of more robust and accurate models.

In this study, we aimed to develop a predictive model for CALs in children with KD and to identify potential risk factors. We employed LASSO regression for feature selection, and the selected variables were subsequently entered into a multivariable logistic regression model, thereby improving the model’s stability and reducing the risk of overfitting. This model may enable the accurate identification of children at higher risk of CALs, facilitate early clinical decision-making, and ultimately improve clinical outcomes.

## Methods

### Study population

In this retrospective analysis, 315 patients diagnosed with KD who were admitted to Children’s Hospital of Hebei Province between January 2024 and May 2025 were screened. After applying the inclusion and exclusion criteria, 255 patients were included in the final analysis, including 75 who developed CAL. The inclusion criteria were as follows: met the 2017 AHA guidelines for complete or incomplete KD [2] and aged ≤ 18 years. The exclusion criteria were as follows: recurrence of KD; receipt of IVIG treatment before admission; no receipt of IVIG treatment during hospitalization; incomplete clinical medical records; and severe infections, chronic diseases, or other complications. All patients with KD received standardized therapy, including IVIG and high-dose aspirin. Clinical type was classified as complete or incomplete KD. Complete KD was defined as the presence of persistent fever (> 38℃) for at least 5 days alongside at least four of the following principal clinical features: polymorphous rash, conjunctival injection, changes in the oral mucosa (such as erythema or fissuring of the lips, strawberry tongue, or pharyngeal injection), peripheral extremity changes, and cervical lymphadenopathy. Incomplete KD was defined as persistent fever (> 38℃) for at least 5 days with fewer than four principal clinical features alongside supportive laboratory findings and/or coronary artery abnormalities identified by echocardiography. IVIG resistance was defined as persistent fever within 36 h after the completion of initial IVIG treatment or recurrence of fever following remission [14]. CALs were diagnosed when the Z-score of any proximal or midsegment coronary artery was ≥ 2.0. The Z-score was calculated using the individual’s height, weight, and coronary artery diameter measured by echocardiography [2].

The study was approved by the Ethics Committee of the Children’s Hospital of Hebei Province (Approval No. 202477). Given the retrospective design of the study, the requirement for informed consent was waived by the ethics committee. This study was conducted in accordance with the Declaration of Helsinki.

### Data collection

Data on demographic characteristics, clinical features, and laboratory indicators were extracted from patients’ electronic medical records before IVIG treatment. Thirty candidate predictors were initially considered. These variables were selected on the basis of prior evidence from the literature, clinical experience, and their routine availability in clinical practice. The variables included sex, age, clinical type, IVIG resistance, white blood cell count (WBC), neutrophil count (NEU), lymphocyte count (LYM), red blood cell count (RBC), hemoglobin (Hb), platelet count (PLT), C-reactive protein (CRP), erythrocyte sedimentation rate (ESR), activated partial thromboplastin time (APTT), prothrombin time (PT), thrombin time (TT), fibrinogen (FIB), immunoglobulin A (IgA), immunoglobulin G (IgG), immunoglobulin M (IgM), alanine aminotransferase (ALT), aspartate aminotransferase (AST), gamma-glutamyl transferase (GGT), total bilirubin (TBIL), direct bilirubin (DBIL), total bile acid (TBA), blood urea nitrogen (BUN), creatinine (Cr), creatine kinase (CK), lactate dehydrogenase (LDH), and hydroxybutyrate dehydrogenase (HBDH). Together, these variables revealed the patients’ inflammatory state, coagulation abnormalities, and immune function.

### Statistical analysis

All analyses were performed using R software (version 4.5.1, The R Foundation for Statistical Computing, Vienna, Austria). Continuous variables were presented as the mean ± standard deviation (SD) for normally distributed data or as the median (interquartile range) for non-normally distributed data. Group comparisons were performed using Student’s *t*-test or the Mann–Whitney *U* test when appropriate. Categorical variables were expressed as frequencies (percentages) and compared using the chi-squared test. Two-sided *P* < 0.05 was considered statistically significant. The proportion of missing data for all variables is presented in Supplementary Table 1. Missing data were handled using multiple imputation by chained equations (MICE) with the “mice” package. Five imputed datasets (m = 5) were generated using predictive mean matching (PMM), with all candidate variables included in the imputation model.

Feature selection was performed using LASSO regression to identify key factors associated with the risk of CALs. The selected variables were then entered into a multivariable logistic regression model. A nomogram was developed to visualize the final prediction model. The discrimination ability of the model was evaluated using the area under the receiver operating characteristic (ROC) curve (AUC), and internal validation was performed using 1,000 bootstrap resamples. Calibration performance was assessed using calibration curves, the Hosmer–Lemeshow goodness-of-fit test, and the Brier score. The Brier score represents the mean squared difference between predicted probabilities and observed outcomes, with lower values indicating better predictive performance. Furthermore, decision curve analysis (DCA) was performed to evaluate the clinical net benefit of the model across a range of threshold probabilities. A sensitivity analysis was conducted to assess the impact of missing data by comparing the predictive performance of models based on complete-case and multiply imputed datasets, as evaluated using ROC curves.

## Results

### Patient characteristics

**Fig. 1:**
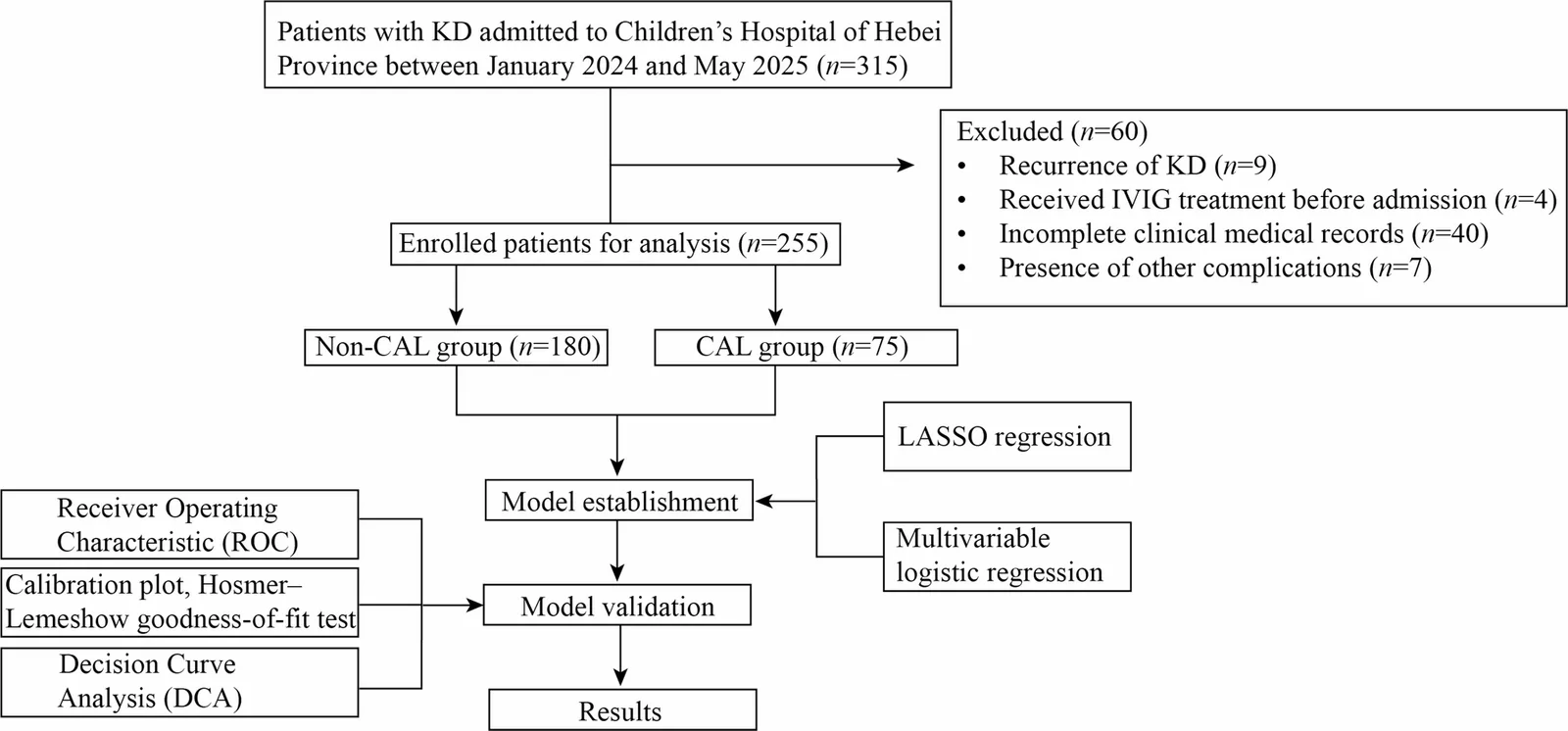
Flow chart of the study design

The flow chart of the study is presented in Fig. 1. In total, 255 patients, including 154 males (60.4%) and 101 females (39.6%), who met the inclusion and exclusion criteria were enrolled, and 217 patients (85.1%) were younger than 5 years. Of the enrolled patients, 75 (29.4%) developed CALs, 28 (11.0%) had incomplete KD, and 19 (7.5%) exhibited IVIG resistance. Significant differences in age, clinical type, LYM, PLT, CRP, TT, FIB, and BUN were observed between children with and without CALs (all *P <* 0.05), whereas no other baseline characteristics differed between these groups (all *P* > 0.05; Table 1).

**Table 1:** Baseline characteristics of patients with KD

Variables	Total (*n* = 255)	Non-CALs (*n* = 180)	CALs (*n* = 75)	*P* value
Sex, *n* (%)				0.535
male	154 (60.4)	106 (58.9)	48 (64.0)	
female	101 (39.6)	74 (41.1)	27 (36.0)	
Age, *n* (%)				< 0.001
< 6 months	18 (7.0)	4 (2.2)	14 (18.7)	
6 months to < 12 months	29 (11.4)	16 (8.9)	13 (17.3)	
12 months to < 5 years	170 (66.7)	133 (73.9)	37 (49.3)	
5 to < 18 years	38 (14.9)	27 (15.0)	11 (14.7)	
Clinical type, *n* (%)				< 0.001
Complete KD	227 (89.0)	170 (94.4)	57 (76.0)	
Incomplete KD	28 (11.0)	10 (5.6)	18 (24.0)	
IVIG resistance, *n* (%)				0.963
No	236 (92.5)	166 (92.2)	70 (93.3)	
Yes	19 (7.5)	14 (7.8)	5 (6.7)	
WBC (×10^9^/L), Median (Q1,Q3)	14.30 (11.45, 17.70)	14.15 (11.47, 17.00)	15.20 (11.50, 19.25)	0.174
NEU (×10^9^/L), Median (Q1,Q3)	9.23 (7.05, 12.17)	9.29 (7.20, 11.91)	9.18 (6.20, 13.23)	0.872
LYM (×10^9^/L), Median (Q1,Q3)	3.31 (2.14, 4.70)	3.07 (1.96, 4.32)	3.80 (2.38, 5.53)	0.005
RBC (×10^12^/L), Median (Q1,Q3)	4.13 (3.92, 4.38)	4.13 (3.94, 4.40)	4.09 (3.86, 4.33)	0.119
Hb (g/L), Mean ± SD	110.57 ± 11.17	111.4 ± 10.06	108.59 ± 13.33	0.103
PLT (×10^9^/L), Median (Q1,Q3)	381.00 (307.50, 451.50)	366.00 (295.75, 430.00)	434.00 (342.50, 533.50)	< 0.001
CRP (mg/L), Median (Q1,Q3)	54.51 (31.99, 83.29)	59.30 (32.02, 87.72)	48.44 (31.09, 67.97)	0.047
ESR (mm/h), Median (Q1,Q3)	67.47 ± 26.96	68.47 ± 25.42	65.08 ± 30.40	0.397
APTT (s), Median (Q1,Q3)	28.60 (26.50, 31.30)	28.40 (26.50, 30.83)	29.00 (26.60, 32.30)	0.172
PT (s), Median (Q1,Q3)	12.00 (11.50, 12.80)	12.00 (11.57, 12.80)	12.00 (11.45, 12.80)	0.816
TT (s), Median (Q1,Q3)	15.80 (15.30, 16.40)	15.65 (15.17, 16.20)	16.20 (15.70, 16.95)	< 0.001
FIB (g/L), Median (Q1,Q3)	4.88 (4.39, 5.73)	5.05 (4.52, 5.80)	4.65 (3.99, 5.46)	0.003
IgA (g/L), Median (Q1,Q3)	0.64 (0.33, 1.00)	0.64 (0.37, 0.99)	0.54 (0.32, 1.00)	0.394
IgG (g/L), Mean ± SD	7.12 (5.38, 9.16)	7.25 (5.54, 9.03)	6.89 (5.10, 9.59)	0.874
IgM (g/L), Median (Q1,Q3)	1.05 (0.74, 1.40)	1.05 (0.76, 1.38)	1.02 (0.74, 1.55)	0.734
ALT (U/L), Median (Q1,Q3)	25.00 (14.00, 80.00)	30.00 (14.00, 98.75)	19.00 (11.00, 56.00)	0.052
AST (U/L), Median (Q1,Q3)	26.00 (20.00, 37.00)	27.00 (21.00, 41.00)	24.00 (19.50, 32.00)	0.095
GGT (U/L), Median (Q1,Q3)	33.00 (13.00, 108.50)	41.00 (14.00, 130.75)	27.00 (13.00, 56.50)	0.078
TBIL (µmol/L), Median (Q1,Q3)	6.10 (4.20, 8.90)	6.20 (4.27, 9.00)	6.10 (4.20, 8.80)	0.938
DBIL (µmol/L), Median (Q1,Q3)	2.70 (1.90, 4.40)	2.70 (2.00, 4.43)	2.50 (1.50, 3.90)	0.083
TBA (µmol/L), Median (Q1,Q3)	6.80 (4.15, 12.80)	6.80 (4.10, 12.43)	7.30 (4.30, 14.30)	0.879
BUN (mmol/L), Median (Q1,Q3)	2.63 (2.00, 3.32)	2.67 (2.18, 3.49)	2.49 (1.79, 3.08)	0.020
Cr (µmol/L), Median (Q1,Q3)	23.00 (19.00, 27.00)	24.00 (19.00, 28.00)	22.00 (19.00, 26.00)	0.081
CK (U/L), Median (Q1,Q3)	34.00 (23.00, 53.00)	35.50 (23.75, 53.00)	33.00 (22.50, 52.00)	0.500
LDH (U/L), Median (Q1,Q3)	244.00 (213.50, 277.00)	246.50 (215.00, 277.25)	235.00 (211.00, 273.00)	0.552
HBDH (U/L), Median (Q1,Q3)	190.00 (169.00, 216.00)	193.50 (169.75, 216.25)	185.00 (169.00, 214.50)	0.324

### Univariate predictive ability and correlation analysis

The ROC curves were plotted for each variable (Supplementary Fig. 1A), and the corresponding AUCs were calculated to evaluate their individual predictive abilities (Supplementary Fig. 1B). Of the included variables, TT featured the highest AUC of 0.695. Thus, all individual factors exhibited limited predictive ability for CAL, making it necessary to develop a model to improve predictive accuracy. Subsequently, we conducted correlation analysis among the included variables, which revealed a high degree of correlation, indicating the presence of multicollinearity (Supplementary Fig. 1C).

### Risk factor screening

**Fig. 2:**
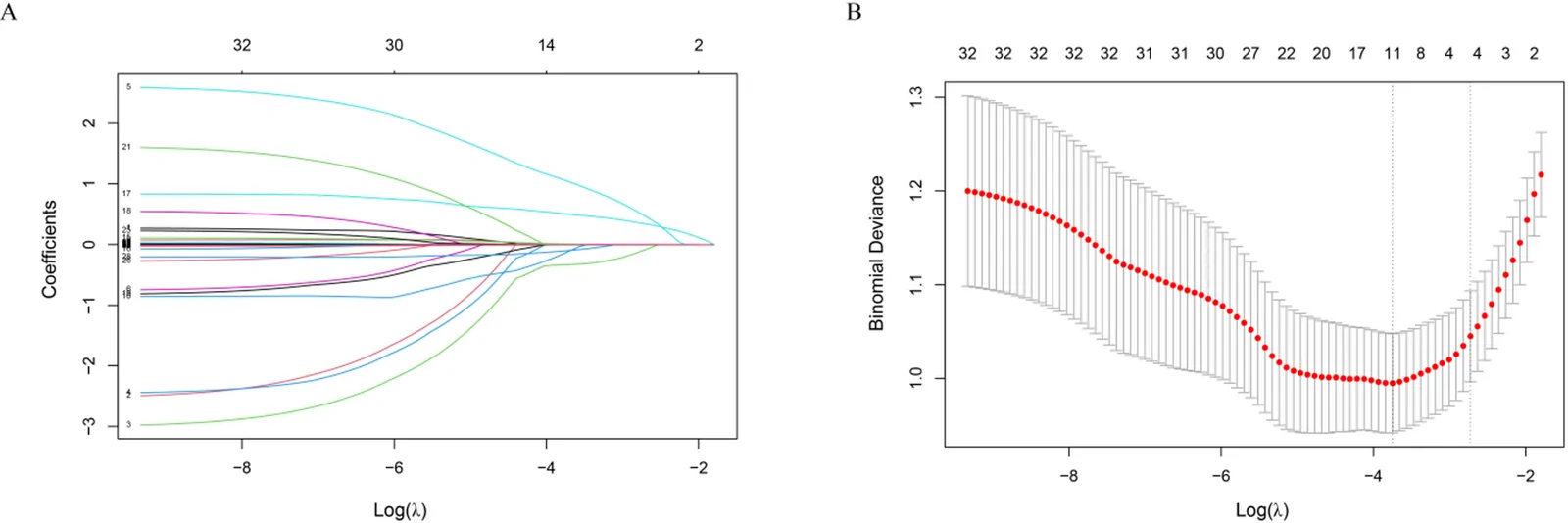
Feature selection based on least absolute shrinkage and selection operator (LASSO) regression. (**A**) LASSO regression coefficient path plot. The x-axis represents the log-transformed regularization parameter (log λ), and the y-axis represents the coefficients of the variables. (**B**) The optimal λ was determined by 10-fold cross-validation. The left vertical dashed line indicates the value of λ that minimizes the cross-validation error (λ.min = 0.0215), whereas the right vertical dashed line corresponds to the value of λ within one standard error of the minimum cross-validation error (λ.1se = 0.0655)

To mitigate potential multicollinearity, LASSO regression was employed for feature selection. Using 10-fold cross-validation, we determined the optimal value of λ (λ.1se = 0.0655) and identified four corresponding variables with non-zero coefficients: age, clinical type, PLT, and TT (Fig. 2A, B). The variables selected by LASSO regression were subsequently incorporated into the multivariable logistic regression analysis. As presented in Table 2, age, clinical type, PLT, and TT were identified as independent risk factors for CAL occurrence. The predictive model was expressed as follows:

logit(P) = − 12.610 − 1.704 × (age 6 months to < 12 months) − 2.202 × (age 12 months to < 5 years) − 2.020 × (age 5 years to < 18 years) + 1.676 × incomplete KD + 0.695 × TT + 0.006 × PLT.

**Table 2:** Multivariable logistic regression analysis of CAL risk in patients with KD

Variables	β	S.E.	OR	95% CI	Z value	*P* value
Age						
< 6 months	Reference					
6 months to < 12 months	-1.704	0.799	0.18	0.038–0.871	-2.134	0.033
12 months to < 5 years	-2.202	0.673	0.11	0.030–0.414	-3.272	0.001
5 to < 18 years	-2.020	0.760	0.13	0.030–0.588	-2.658	0.008
Clinical type						
Complete KD	Reference					
Incomplete KD	1.676	0.501	5.34	2.002–14.267	3.348	0.001
TT (s)	0.695	0.189	2.00	1.383–2.901	3.673	< 0.001
PLT (×10^9^/L)	0.006	0.002	1.01	1.002–1.010	3.828	< 0.001

### Construction and evaluation of the prediction model

**Fig. 3:**
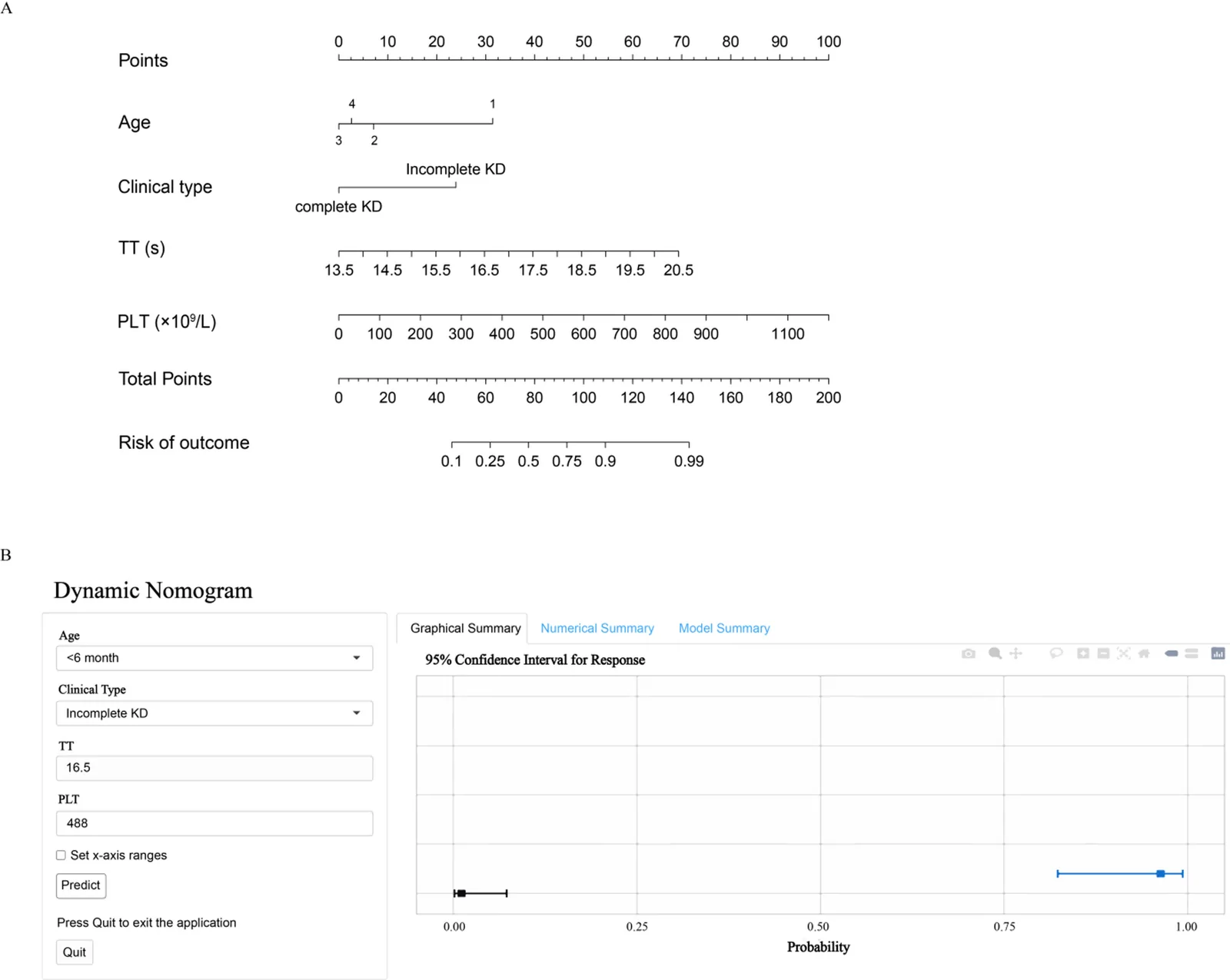
(**A**) Nomogram for predicting the risk of coronary artery lesions (CALs) in children with Kawasaki disease. The variable “age” was categorized as follows: 1, < 6 months; 2, 6 months to < 12 months; 3, 12 months to < 5 years; and 4, 5 years to < 18 years. (**B**) The web-based dynamic nomogram

Based on the results of the multivariable logistic regression analysis, a nomogram incorporating four independent risk factors was developed to predict the risk of CALs in children with KD. As depicted in Fig. 3A, a 4-year-old patient (0 points) diagnosed with incomplete KD (22.5 points), with a TT of 16.2 s (25.0 points), and a PLT of 585 × 10^9^/L (47.5 points) has a total score of 95.0 points, corresponding to an approximate predicted probability of 82.3% for the development of CALs. A web-based dynamic nomogram was developed, which can be accessed at https://kd-cal-prediction.shinyapps.io/dynnomapp/ (Fig. 3B).

**Fig. 4:**
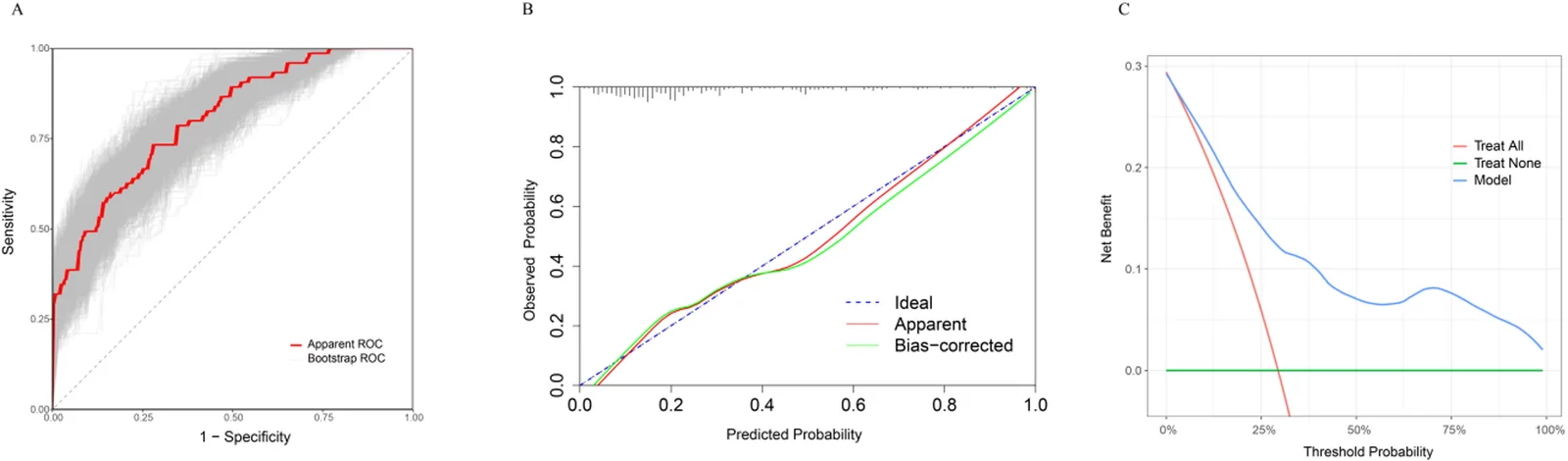
Performance of the prediction model. (**A**) Receiver operating characteristic curves after internal validation using bootstrap resampling (1,000 replicates). (**B**) Calibration curve of the model. The x-axis represents the predicted probability of CALs derived from the model, whereas the y-axis represents the observed incidence of CALs. (**C**) Decision curve analysis. The blue line denotes the net benefit of the prediction model across threshold probabilities. The “treat all” strategy assumes that all patients at high risk for CALs would receive the intervention, whereas the “treat none” strategy assumes that no patients receive additional management

An ROC curve was used to evaluate the ability of the model to distinguish between patients at low and high risk of CALs. Following internal validation with 1,000 bootstrap resamples, the ROC curve exhibited an AUC of 0.807 (95% CI = 0.750–0.860), which indicated that the model exhibited good ability to discriminate patient risk (Fig. 4A). Based on our calibration curve, we identified a calibration intercept of 0.000 (95% CI = − 0.327–0.316), a calibration slope of 1.000 (95% CI = 0.721–1.318), good model fit according to the Hosmer–Lemeshow goodness-of-fit test (*χ*^2^ = 5.869, *P* = 0.662), and a Brier score of 0.149, all indicating good overall accuracy of the predicted probabilities (Fig. 4B). DCA illustrated that predicting the risk of CALs might provide clinical benefit for patients with CALs when the threshold probability exceeds 2% (Fig. 4C). The sensitivity analysis demonstrated consistent results between the complete-case and multiple imputation methodologies, indicating that the model performance was robust to missing data handling (Supplementary Fig. 2).

## Discussion

The incidence of KD has been steadily increasing in recent years. Although KD was initially considered an acute, self-limited illness of childhood, it is now recognized to be associated with an increased risk of cardiovascular complications that may extend into adulthood [15, 16]. Therefore, identifying risk factors for CALs in children with KD and establishing a model for early prediction are of significant clinical importance for KD management. In our cohort, the incidence of CALs in children with KD reached 29.4% (75/255), which was higher than that reported in previous studies [17, 18]. This relatively high incidence might be explained by several factors. First, the diagnostic criteria for CALs strictly followed the AHA guidelines, which include mild coronary artery dilatation, thereby increasing the likelihood of detecting coronary artery abnormalities. Second, most patients in this cohort underwent serial echocardiographic assessments during the acute, subacute, and follow-up phases, permitting the identification of transient or mild coronary artery changes that might be missed with a single examination. Third, as a tertiary pediatric referral center, our hospital might receive a higher proportion of patients with more severe disease, potentially resulting in referral bias. In addition, variations in patient demographics, such as age distribution and the proportion of patients with incomplete KD, might also have contributed to differences in the reported incidence of CALs across studies. These factors may affect the overall predictive performance of the model when applied to external populations. Therefore, external validation in independent cohorts is necessary to confirm the robustness and clinical applicability of the model. Meanwhile, among the 255 patients with KD, 19 (7.5%) were resistant to IVIG, which is consistent with findings from previous studies [19–23].

We employed commonly used demographic and laboratory indicators as candidate predictors and ultimately developed the predictive model using variables selected through a combination of traditional statistical methods and modern ML algorithms. LASSO regression, by introducing L1 regularization, effectively addressed high-dimensional data and reduced the risk of overfitting by shrinking the coefficients of irrelevant features toward zero [24]. Based on this approach, age, clinical type, TT, and PLT were identified as key predictors and subsequently included in the multivariable logistic regression model. Furthermore, internal validation using bootstrap resampling confirmed that the model exhibited good discrimination (AUC = 0.807) and calibration. In addition, DCA indicated that the model provides net clinical benefits across a wide range of threshold probabilities. These findings suggest that the model may have potential clinical utility in assisting clinicians with preventive and interventional decision-making. Specifically, patients with KD identified as having a high risk of CALs might benefit from intensified echocardiographic surveillance and closer clinical monitoring during the acute phase. In addition, early recognition of high-risk patients may help clinicians consider more aggressive therapeutic strategies when clinically appropriate. Conversely, patients with a lower predicted risk could be managed with routine follow-up and standard monitoring, thereby avoiding unnecessary examinations or interventions. Such risk-stratified management may help optimize the allocation of medical resources and support more individualized clinical decision-making.

Several predictive models for CALs in patients with KD have been previously reported; however, the predictors and model performance vary across studies. For example, Hu et al. [25] developed a nomogram based on laboratory indicators, including sodium, Hb, PLT, D-dimer, and cystatin C levels, achieving good discriminative ability with AUCs of 0.819 and 0.844 in the training and validation sets, respectively. In another large-scale study including 6,847 children, Li et al. [18] constructed a model incorporating sex, IVIG non-response, Hb, platelet distribution width, PLT, and albumin, with AUCs of 0.671 and 0.703 in the training and validation cohorts, respectively. In the present study, we identified age, clinical type, TT, and PLT as independent predictors of CALs in children with KD. Compared with previous models that primarily relied on multiple laboratory parameters, our model incorporated both clinical characteristics and coagulation or hematologic indicators. This combination may provide complementary information reflecting disease severity, inflammatory responses, and coagulation abnormalities associated with vascular injury in KD. Notably, the predictors included in our model are routinely available clinical or laboratory indicators that can be readily obtained during the early stage of hospitalization, which may facilitate the implementation of the model in routine clinical practice.

The pathogenesis underlying the progression of CALs in patients with KD remains incompletely understood. Orenstein et al. identified three distinct but interconnected pathological processes involved in KD vasculopathy [26]. Necrotizing arteritis typically develops within the first 2 weeks after KD onset, with neutrophil infiltration into the walls of small- and medium-sized arteries, particularly the coronary arteries, leading to an increased risk of coronary artery aneurysm and thrombosis [27]. During subacute or chronic vasculitis, immune cells such as lymphocytes, monocytes, and macrophages infiltrate the arterial wall and release multiple proinflammatory cytokines [28, 29]. Among these cytokines, interleukin-6 induces vascular endothelial cell injury and exposure of subendothelial collagen, which facilitates platelet adhesion and activation, ultimately resulting in thrombocytosis. Activated platelets interact with leukocytes to form aggregates, which in turn release inflammatory mediators that further aggravate vascular endothelial injury [30, 31]. The final pathological process, characterized by luminal myofibroblastic proliferation, contributes substantially to long-term vascular remodeling and cardiovascular complications [26]. Taken together, platelets play an important role in the inflammatory immune response during KD pathogenesis and contribute to the progression of CALs.

Thrombocytosis is a characteristic feature of KD that typically develops during the second to third week of illness. This period coincides with the time when coronary artery aneurysms most commonly appear [2, 32]. Several scoring systems have incorporated PLT and other laboratory parameters to identify children with KD at low or high risk of coronary artery complications and IVIG resistance [14, 33]. Kocatürk et al. reported the upregulation of platelet-related genes using transcriptomic data derived from the whole blood of patients with acute KD and further confirmed that the development of cardiovascular lesions is associated with increased PLT in the *Lactobacillus casei* cell wall extract murine model of KD vasculitis [34]. A large prospective study involving 6,847 children with KD identified PLT as an independent risk factor for CALs development [18]. Consistent with these findings, our data further revealed that children in the CALs group had higher PLT levels than those in the non-CALs group, supporting the hypothesis that elevated PLT in children with KD denotes an increased risk of CALs.

In addition, our study found that patients with incomplete KD had a higher risk of CALs, consistent with previous findings [35]. One possible explanation is that incomplete KD often presents with atypical clinical manifestations, leading to diagnostic uncertainty and delayed IVIG treatment, thereby increasing the risk of developing coronary artery complications.

This study had several limitations. First, the retrospective nature of the study might have introduced selection and information bias during data collection, which cannot be completely eliminated. To minimize these biases, we implemented rigorous inclusion and exclusion criteria to ensure that the cases included in this study were representative. Second, the model incorporated only a limited set of predictors. Integrating additional clinical manifestations and newly identified predictive factors, particularly immuno-inflammatory–related laboratory indicators, in future studies will be essential to further enhance the comprehensiveness of the prediction model. Third, although the internal validation using bootstrap resampling demonstrates that the model performs well in predicting CAL in patients with KD, the absence of external validation restricts the generalizability and robustness of the model.

Therefore, future multicenter studies with larger sample sizes are necessary to further validate the reliability and generalizability of the model. In addition, prospective impact studies are needed prior to clinical implementation to determine whether the use of this model can improve clinical decision-making and patient outcomes.

## Conclusions

This study identified age, clinical type, TT, and PLT as independent predictors of CALs in patients with KD. Based on these risk factors, we developed a prediction model with good discrimination, calibration, and clinical utility. The model aims to facilitate the early identification of high-risk patients and the implementation of timely and effective interventions, ultimately reducing the incidence of severe cardiovascular complications.

## Supplementary Information

Below is the link to the electronic supplementary material.

Supplementary Material 1

## Data Availability

The datasets used and/or analysed during the current study are available from the corresponding author on reasonable request.

## Abbreviations

KD: Kawasaki disease
CAL: Coronary artery lesions
IVIG: Intravenous immunoglobulin
LASSO: Least absolute shrinkage and selection operator
ROC: Receiver operating characteristic curves
DCA: Decision curve analysis
AUC: Area under ROC curve
AHA: American Heart Association
ML: Machine learning

## References

[1] Uehara R, Belay ED. Epidemiology of Kawasaki Disease in Asia, Europe, and the United States. J Epidemiol. 2012;22:79–85. 10.2188/jea.JE20110131. 22307434 10.2188/jea.JE20110131 PMC3798585

[2] McCrindle BW, Rowley AH, Newburger JW, et al. Diagnosis, Treatment, and Long-Term Management of Kawasaki Disease: A Scientific Statement for Health Professionals From the American Heart Association. Circulation. 2017;135:e927–99. 10.1161/CIR.0000000000000484. 28356445 10.1161/CIR.0000000000000484

[3] Makino N, Nakamura Y, Yashiro M, et al. Nationwide epidemiologic survey of Kawasaki disease in Japan, 2015–2016. Pediatr Int. 2019;61:397–403. 10.1111/ped.13809. 30786118 10.1111/ped.13809

[4] Maddox RA, Person MK, Kennedy JL, et al. Kawasaki Disease and Kawasaki Disease Shock Syndrome Hospitalization Rates in the United States, 2006–2018. Pediatr Infect Disease J. 2021;40:284–8. 10.1097/INF.0000000000002982. 33264213 10.1097/INF.0000000000002982

[5] Friedman KG, Gauvreau K, Hamaoka-Okamoto A, et al. Coronary Artery Aneurysms in Kawasaki Disease: Risk Factors for Progressive Disease and Adverse Cardiac Events in the US Population. J Am Heart Assoc. 2016;5:e003289. 10.1161/JAHA.116.003289. 27633390 10.1161/JAHA.116.003289 PMC5079009

[6] Kuo H-C. Preventing coronary artery lesions in Kawasaki disease. Biomed J. 2017;40:141–6. 10.1016/j.bj.2017.04.002. 28651735 10.1016/j.bj.2017.04.002 PMC6136281

[7] Newburger JW, Takahashi M, Burns JC, Kawasaki, Disease. J Am Coll Cardiol. 2016;67:1738–49. 10.1016/j.jacc.2015.12.073. 27056781 10.1016/j.jacc.2015.12.073

[8] Wang R, Wei D, Gu M, et al. Nomogram model based on serum chitotriosidase activity to predict coronary artery aneurysm in Kawasaki disease. Coron Artery Dis. 2025;36:510–4. 10.1097/MCA.0000000000001492. 39751641 10.1097/MCA.0000000000001492

[9] Wang Y, Lin Y, Zhang L, et al. Lymphocyte-C-reactive protein ratio combined with albumin upon admission predicts coronary artery dilation and aneurysm formation in pediatric patients with Kawasaki disease: a retrospective cohort study. Expert Rev Clin Immunol. 2024;20:1127–33. 10.1080/1744666X.2024.2385765. 39072430 10.1080/1744666X.2024.2385765

[10] Zhang H. Expression of oxidative stress and inflammatory indicators for coronary artery disease in kawasaki disease. Mediterr J Hematol Infect Dis. 2024;16:e2024052. 10.4084/MJHID.2024.052. 38984102 10.4084/MJHID.2024.052 PMC11232689

[11] Zhou X, Peng Y, Yi Q. A nomogram model for predicting recurrent coronary thrombosis in kawasaki disease patients. J Inflamm Res. 2025;18:105–14. 10.2147/JIR.S473511. 39780986 10.2147/JIR.S473511 PMC11705992

[12] Bunea F, She Y, Ombao H, et al. Penalized least squares regression methods and applications to neuroimaging. NeuroImage. 2011;55:1519–27. 10.1016/j.neuroimage.2010.12.028. 21167288 10.1016/j.neuroimage.2010.12.028 PMC5485905

[13] Rajkomar A, Dean J, Kohane I. Machine Learning in Medicine. N Engl J Med. 2019;380:1347–58. 10.1056/NEJMra1814259. 30943338 10.1056/NEJMra1814259

[14] Kobayashi T, Inoue Y, Takeuchi K, et al. Prediction of Intravenous Immunoglobulin Unresponsiveness in Patients With Kawasaki Disease. Circulation. 2006;113:2606–12. 10.1161/CIRCULATIONAHA.105.592865. 16735679 10.1161/CIRCULATIONAHA.105.592865

[15] Daniels LB, Roberts S, Moreno E, et al. Long-term health outcomes in young adults after Kawasaki disease. Int J Cardiol Heart Vasc. 2022;40:101039. 10.1016/j.ijcha.2022.101039. 35573651 10.1016/j.ijcha.2022.101039 PMC9096130

[16] Gordon JB, Daniels LB, Kahn AM, et al. The Spectrum of Cardiovascular Lesions Requiring Intervention in Adults After Kawasaki Disease. JACC: Cardiovasc Interventions. 2016;9:687–96. 10.1016/j.jcin.2015.12.011. 10.1016/j.jcin.2015.12.011 27056307

[17] Gong C, Su Z, Li Q, et al. A risk stratification model for coronary artery lesions in kawasaki disease: Focus on subgroup-specific utility. Front Cardiovasc Med. 2025;12:1543767. 10.3389/fcvm.2025.1543767. 40364829 10.3389/fcvm.2025.1543767 PMC12069354

[18] Li C, Zhang H, Yin W, et al. Development and validation of a nomogram-based prognostic model to predict coronary artery lesions in Kawasaki disease from 6847 children in China. Comput Methods Programs Biomed. 2025;260:108588. 10.1016/j.cmpb.2025.108588. 39793529 10.1016/j.cmpb.2025.108588

[19] Wang Y, Cao Y, Li Y, et al. Development of an immunoinflammatory indicator-related dynamic nomogram based on machine learning for the prediction of intravenous immunoglobulin-resistant Kawasaki disease patients. Int Immunopharmacol. 2024;134:112194. 10.1016/j.intimp.2024.112194. 38703570 10.1016/j.intimp.2024.112194

[20] Huang H, Jiang J, Shi X, et al. Nomogram to predict risk of resistance to intravenous immunoglobulin in children hospitalized with Kawasaki disease in Eastern China. Ann Med. 2022;54:442–53. 10.1080/07853890.2022.2031273. 35099338 10.1080/07853890.2022.2031273 PMC8812733

[21] Yang S, Song R, Zhang J, et al. Predictive tool for intravenous immunoglobulin resistance of Kawasaki disease in Beijing. Arch Dis Child. 2019;104:262–7. 10.1136/archdischild-2017-314512. 30026252 10.1136/archdischild-2017-314512

[22] Shao S, Zhou K, Liu X, et al. Predictive Value of Serum Lipid for Intravenous Immunoglobulin Resistance and Coronary Artery Lesion in Kawasaki Disease. J Clin Endocrinol Metabolism. 2021;106:e4210–20. 10.1210/clinem/dgab230. 10.1210/clinem/dgab230 33837779

[23] Deng L, Zhao J, Wang T, et al. Construction and validation of predictive models for intravenous immunoglobulin–resistant kawasaki disease using an interpretable machine learning approach. Clin Exp Pediatr. 2024;67:405–14. 10.3345/cep.2024.00549. 39048087 10.3345/cep.2024.00549 PMC11298769

[24] Kamkar I, Gupta SK, Phung D, et al. Stabilizing l 1 -norm prediction models by supervised feature grouping. J Biomed Inform. 2016;59:149–68. 10.1016/j.jbi.2015.11.012. 26689771 10.1016/j.jbi.2015.11.012

[25] Hu C, Yan X, Song H, et al. Establishment and validation of a nomogram for coronary artery lesions in children with kawasaki disease. Front Cardiovasc Med. 2025;11:1522473. 10.3389/fcvm.2024.1522473. 39877016 10.3389/fcvm.2024.1522473 PMC11772273

[26] Orenstein JM, Shulman ST, Fox LM, et al. Three Linked Vasculopathic Processes Characterize Kawasaki Disease: A Light and Transmission Electron Microscopic Study. PLoS ONE. 2012;7:e38998. 10.1371/journal.pone.0038998. 22723916 10.1371/journal.pone.0038998 PMC3377625

[27] Takahashi K, Oharaseki T, Yokouchi Y. Histopathological aspects of cardiovascular lesions in Kawasaki disease. Int J Rheum Dis. 2018;21:31–5. 10.1111/1756-185X.13207. 29105353 10.1111/1756-185X.13207

[28] Noval Rivas M, Arditi M. Kawasaki disease: pathophysiology and insights from mouse models. Nat Rev Rheumatol. 2020;16:391–405. 10.1038/s41584-020-0426-0. 32457494 10.1038/s41584-020-0426-0 PMC7250272

[29] Noval Rivas M, Kocatürk B, Franklin BS, et al. Platelets in Kawasaki disease: mediators of vascular inflammation. Nat Rev Rheumatol. 2024;20:459–72. 10.1038/s41584-024-01119-3. 38886559 10.1038/s41584-024-01119-3

[30] Straface E, Gambardella L, Metere A, et al. Oxidative stress and defective platelet apoptosis in naïve patients with Kawasaki disease. Biochem Biophys Res Commun. 2010;392:426–30. 10.1016/j.bbrc.2010.01.040. 20079717 10.1016/j.bbrc.2010.01.040

[31] Jin J, Wang J, Lu Y, et al. Platelet-Derived Microparticles: A New Index of Monitoring Platelet Activation and Inflammation in Kawasaki Disease. Indian J Pediatr. 2019;86:250–5. 10.1007/s12098-018-2765-2. 30159809 10.1007/s12098-018-2765-2

[32] Arora K, Guleria S, Jindal AK, et al. Platelets in Kawasaki disease: Is this only a numbers game or something beyond? Genes Dis. 2020;7:62–6. 10.1016/j.gendis.2019.09.003. 32181276 10.1016/j.gendis.2019.09.003 PMC7063415

[33] Egami K, Muta H, Ishii M, et al. Prediction of resistance to intravenous immunoglobulin treatment in patients with Kawasaki disease. J Pediatr. 2006;149:237–40. 10.1016/j.jpeds.2006.03.050. 16887442 10.1016/j.jpeds.2006.03.050

[34] Kocatürk B, Lee Y, Nosaka N, et al. Platelets exacerbate cardiovascular inflammation in a murine model of Kawasaki disease vasculitis. JCI Insight. 2023;8:e169855. 10.1172/jci.insight.169855. 37279077 10.1172/jci.insight.169855 PMC10443810

[35] Gao Y, Peng L, Liu J, et al. Establishment and validation of a nomogram for predicting intravenous immunoglobulin resistance and coronary artery lesion involvement in Kawasaki disease: a retrospective study. Clin Rheumatol. 2025;44:799–809. 10.1007/s10067-025-07321-2. 39808233 10.1007/s10067-025-07321-2

