# Supplementary material for: Predicting coronary artery lesions in Kawasaki disease: a nomogram based on LASSO regression feature selection
Source: BMC Med Inform Decis Mak. 2026 Jun 16;26:323. doi: 10.1186/s12911-026-03637-4 (PMC13505040; doi:10.1186/s12911-026-03637-4)
Supplement: Supplementary file 1 [file 12911_2026_3637_MOESM1_ESM.docx]

**Supplementary Table 1.** The number of missing data

| **Variable** | **Amount of missing** | |
| --- | --- | --- |
|  | ***n*** | **%** |
| NEU | 2 | 0.78 |
| LYM | 2 | 0.78 |
| RBC | 4 | 1.57 |
| ESR | 10 | 3.92 |
| APTT | 12 | 4.71 |
| PT | 12 | 4.71 |
| TT | 12 | 4.71 |
| FIB | 12 | 4.71 |
| IgA | 7 | 2.75 |
| IgG | 7 | 2.75 |
| IgM | 7 | 2.75 |
| ALT | 2 | 0.78 |
| AST | 2 | 0.78 |
| GGT | 2 | 0.78 |
| TBIL | 3 | 1.18 |
| DBIL | 3 | 1.18 |
| TBA | 2 | 0.78 |
| BUN | 2 | 0.78 |
| Cr | 2 | 0.78 |
| CK | 4 | 1.57 |
| LDH | 4 | 1.57 |
| HBDH | 4 | 1.57 |

**Supplementary Figure 1.** (A) ROC curves for all variables. (B) The AUC for each variable. (C) Correlation heatmap of the analyzed variables. Blue indicates positive correlations, whereas red indicates negative correlations. The circle size reflects the strength of the correlation, with larger circles indicating stronger correlations (**P* < 0.05, ***P* < 0.01, ****P* < 0.001).

**Supplementary Figure 2.** Sensitivity analysis before and after multiple imputation.
